# Patient Usage of Apps to Access Online Medical Records

**DOI:** 10.1001/jamanetworkopen.2023.43312

**Published:** 2023-11-14

**Authors:** Wesley Barker, Chelsea Richwine

**Affiliations:** 1 Office of the National Coordinator for Health IT, US Department of Health and Human Services, Washington, DC

## Abstract

This cross-sectional study analyzes the use of patient portal apps and third-party apps for managing multiple patient portals between 2019 and 2022.

## Introduction

US health care’s rapid digitization over the past decade has enabled broad patient electronic access to their online medical records.^1^ The federal government facilitated this access through the certification of electronic health records (EHRs). Initially, patient portals, which provide web-based or app-based patient access, enabled patients to view, download, and transmit medical records.^2^ More recently, patient access has been enabled through secure, third-party app connections that use a standards-based application programming interface (API), providing patients the ability to access and combine multiple online records in 1 app.^3^ This cross-sectional study used data collected from an app store and the Health Information National Trends Survey (HINTS 6) to analyze patient use of apps to access health information, including traditional patient portal apps available from leading EHRs and emerging third-party apps.

## Methods

This cross-sectional study followed STROBE reporting guidelines and did not require institutional review board approval or participant informed consent because it was not human participant research in accordance with 45 CFR 46 and used publicly available data. We wrote a computer program using R statistical software version 4.2.1 (R Project for Statistical Computing) to collect app installation and review count data on 26 patient portal apps and 22 third-party apps (eAppendix 1 in Supplement 1) from the Google Play website in 2019, 2021, and 2022. We selected these apps based on market research and the market share for relevant EHR, insurer, and health care organization portal apps included in the study. We selected the app store because it publicly provides installation data and represents about one-half of the US mobile market. We complemented the app store data with data from HINTS 6, a nationally representative survey of US adults, fielded March to November 2022 (eAppendix 2 in Supplement 1). Bivariate analyses of HINTS data were conducted using Stata/SE statistical software version 15 (StataCorp). Differences in outcomes were assessed using χ^2^ tests of independence, and a 2-sided *P* < .05 was considered statistically significant. All analyses of HINTS data used survey weighting procedures (weighted percentages reported in the Results) with jackknife replicate weights to account for the complex survey design. Data analysis was conducted from March to April 2023.

## Results

The HINTS study population comprised 6252 adults (mean [SE] age 48.8 [0.2] years; 3511 female respondents [50%]; 3528 White respondents [61%]) including 4690 college-educated respondents [71%] and 5441 respondents [88%] living in an urban area. According to the HINTS, in 2022, 1733 of 3627 individuals (50%) who reported accessing their portal did so via an app, and 2011 of 4578 individuals (44%) reported having multiple portals. According to the app store data, in 2022, patient portal apps, including those developed by EHR developers (eg, Epic) and health care organizations and insurers (eg, Kaiser Permanente) comprised almost 99% of app installations (43 349 252 of 43 922 929 installations [unweighted percentage, 98.7%]) and third-party apps accounted for just over 1% of all installations (573 677 of 43 922 929 installations [unweighted percentage, 1.3%]) (Table). We observed a large increase in third-party app use in 2021 and 2022, however, CommonHealth represented 77% (unweighted) of those installations (350 705 of 454 381 installations).

**Table.** Patient Portal and Third-Party Patient Access App Installations and Reviews, 2019-2022^a^

Year	Application installations and reviews, No./Total No. (%)	
	Patient portal apps	Third-party apps^b^
**Installations**		
2019	17 159 602/17 188 198 (99.8)	28 596/17 188 198 (0.2)
2021	35 973 460/36 427 841 (98.8)	454 381/36 427 841 (1.2)
2022	43 349 252/43 922 929 (98.7)	573 677/43 922 929 (1.3)
**Reviews**		
2019	214 880/215 133 (99.9)	253/215 133 (0.1)
2021	361 107/363 383 (99.4)	2276/363 383 (0.6)
2022	477 442/484 177 (98.6)	6735//484 177 (1.4)

^a^ A total of 22 unique patient portal apps and 26 unique third-party apps were included in the analysis.

^b^ One third-party app, CommonHealth, comprised 350 705 (77%) of third-party app installations and 1505 (66%) reviews in 2021 and 444 119 (77%) of installations and 5790 (86%) reviews in 2022.

Similarly, in the HINTS, 104 individuals (2%) reported they used a third-party app (eg, CommonHealth or Apple Health) to combine their health information from multiple patient portals (Figure). Although most individuals had only 1 patient portal (2567 of 4578 respondents [56%]), 1907 of 4578 respondents (41%) reported having multiple portals but did not use a third-party app to organize information from these portals.

**Figure.**
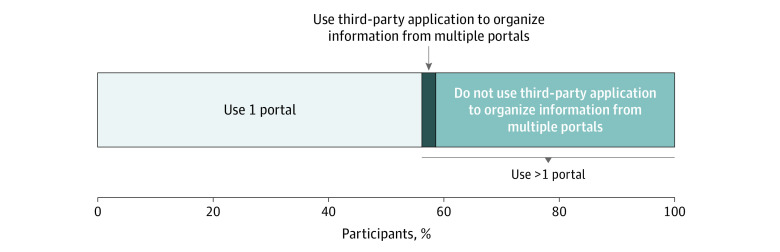
Percentages of Individuals With Multiple Patient Portals and Use of Third-Party Apps, 2022 The horizontal stacked bar chart reports the share of individuals who reported having 1 (2567 individuals [56%]) vs >1 (2011 individuals [44%]) patient portal or online medical record. Individuals without any online medical records or patient portals were excluded from the denominator. Of the 2011 individuals with multiple portals, 104 (2%) reported using a third-party app (eg, Apple Health Records or CommonHealth) to combine their medical information from these different portals into one place and 1907 (41%) did not use a third-party app for this purpose. The percentages of app use vs no app use in the bar chart do not sum to 44% due to rounding. The data come from the Health Information National Trends Survey, 2022.

## Discussion

This cross-sectional study found that third-party apps represented an emerging method for patients to access their health information, but their current use was low compared with patient portal apps.^4,5^ However, 44% of individuals reported having multiple patient portals in 2022, suggesting there may be demand for apps that can be used to organize information from multiple portals into a single location. EHR developers were federally required to update and provide the new standards-based APIs by December 31, 2022, so these data show the state of patient access before this new policy was fully implemented. We will continue to track these data to assess the impact of the policy. The study only included app install data from a single app store, limiting analysis to installs and not use of the apps. We also lacked data on app users and could not generalize the results across demographic characteristics. As federal policies continue to broaden access and provide more choices to patients, it will be important to educate individuals on how they can effectively manage their health information and make informed decisions about their health.^6^

## Notes

Supplement 1. **eAppendix 1.** App Store Apps **eAppendix 2.** HINTS Survey Questions and Response Options

Supplement 2.
Data Sharing Statement
