# Supplementary material for: Patient Usage of Apps to Access Online Medical Records
Source: JAMA Netw Open. 2023 Nov 14;6(11):e2343312. doi: 10.1001/jamanetworkopen.2023.43312 (PMC10646724; doi:10.1001/jamanetworkopen.2023.43312)
Supplement: Supplement 1. — eAppendix 1. App Store Apps eAppendix 2. HINTS Survey Questions and Response Options [file jamanetwopen-e2343312-s001.pdf]

Supplemental Online Content

Barker W, Richwine C. Patient usage of apps to access online medical records. *JAMA Netw Open*. 2023;6(11):e2343312. doi: 10.1001/jamanetworkopen.2023.43312

eAppendix 1. App Store Apps

eAppendix 2. HINTS Survey Questions and Response Options

This supplemental material has been provided by the authors to give readers additional information about their work.

© 2023 Barker W et al. *JAMA Network Open*

eAppendix 1. App Store Apps

Patient Portal Apps

Note: Source URL = https://play.google.com/store/apps/details

App Developer	Google Play App
Allscripts	id=com.jardogs.fmhmobile
Cerner Corporation	id=com.cerner.iris.play
Banner Health	id=com.bannerhealth.BannerHealthMobileApp
Dignity Health	id=org.dignityhealth.healthelife.chw_ca.play
Ascension Health	id=org.ascension.android.ascn.onlinecare
Trinity Health	id=org.trinityhealth.trinityhealth
Get Real Health	id=com.yourlifeyourdata.lydia
InteliChart	id=ic.mobile.pap
eClinicalWorks	id=com.ecw.healow
Epic Systems Corporation	id=epic.mychart.android
Mayo Clinic	id=com.mayoclinic.patient
Partners Healthcare	id=org.partners.ppgmob
UC Health	id=com.uchealth.mobile
Wellspan Health	id=com.wellspan.wellspanmobileapp
UPMC	id=com.upmc.enterprises.myupmc
Sutter Health	id=org.sutterhealth.myhealthonline
Mercy Health	id=net.mercy.mymercy
Kaiser Permanente	id=org.kp.m
MEDHOST	id=com.medhost.app
MEDITECH	id=com.meditech.PatientPhm
NextGen	id=com.nextmd.nextgenpp
Quest Diagnostics Incorporated	id=com.myquest
Laboratory Corporation	id=com.labcorp.patientportal
Medfusion	id=com.medfusion.claire
SymphonyCare	id=com.symphony.symphonycare
Health Companion	id=com.stabilix.hcandroid.activity

Third-Party Apps

Note: Source URL = https://play.google.com/store/apps/details

App Developer	Google Play App
1uphealth	id=health.OneUp.OneUpHealth
Andaman7	id=com.andaman7.android
b.well	id=com.icanbwell.members
Backpack Health	id=com.backpackhealth.backpack
Care Evolution	id=com.careevolution.myfhr
Coral Health Research and Discovery	id=com.mycoralhealth.coralhealth
Curamei Technologies LLC	id=com.curameitech.app

© 2023 Barker W et al. *JAMA Network Open*

drowl	id=com.mrowl.androiddrowl
Get Real Health	id=com.yourlifeyourdata.lydia
Giffen Solutions	id=com.giffen.medxvault
Health Gorilla	id=com.healthgorilla.doctors
Health Wizz	id=com.wellness.healthwizz.gaming&hl=en_US
HealthHive	id=com.healthhive
HealthLynked Corp.	id=com.healthlynked
Humetrix	id=com.humetrix.iBlueButton
Innovaccer Inc	id=com.innovaccer.inhealth
MaxMD	id=patient.app.maxmd.org.maxmdapp
Medlio	id=com.medl.io.patientapp
mpowered health	id=com.mpoweredhealth
onerecord	id=com.onerecord.OneRecord
PaxeraHealth Corp	id=com.paxeramed.RadPassport&hl=en
The Commons Project Foundation	id=org.thecommonsproject.android.phr

eAppendix 2. HINTS Survey Questions and Response Options

E3. How many times did you access your online medical record or patient portal in the last 12 months?

- I do not have an online medical record or patient portal that was offered to me by a health care provider or insurer
- 0
- 1 to 2 times
- 3 to 5 times
- 6 to 9 times
- 10 or more times

E4. How did you access your online medical record or patient portal?

- App
- Website
- Both app and website
- Don’t know

*Note: Only includes respondents who reported accessing their online medical record or patient portal in the past 12 months (**E3**). In the manuscript, “App” and “Both app and website” were combined to identify the share of individuals who accessed their online medical records via an app.*

E8. Do you have one, or more than one patient portal or online medical record?

- One
- More than one

E9. Have you ever used an app like 'Apple Health Records' or 'CommonHealth' to combine your medical information from different patient portals or online medical records into one place?

- Yes
- No

Note: Only includes respondents who indicated they had "More than one" patient portal or online medical record in question ***E8****.*

Source: [HINTS 6 (2022)]

© 2023 Barker W et al. *JAMA Network Open*
