# Supplementary material for: Patient Usage of Apps to Access Online Medical Records
Source: JAMA Netw Open. 2023 Nov 14;6(11):e2343312. doi: 10.1001/jamanetworkopen.2023.43312 (PMC10646724; doi:10.1001/jamanetworkopen.2023.43312)
Supplement: Supplement 2. — Data Sharing Statement [file jamanetwopen-e2343312-s002.pdf]

Data Sharing Statement

Barker. Patient Usage of Apps to Access Online Medical Records. *JAMA Netw Open*.
Published November 14, 2023. doi:10.1001/jamanetworkopen.2023.43312

Data
Data available: Yes
Data types: Data (not involving human participants), Data dictionary
How to access data: Data for HINTS 6 is publicly available through its website: https://hints.cancer.gov/. Other data used is available upon request through corr author: wesley.barker@hhs.gov.
When available: With publication

Supporting Documents
Document types: None

Additional Information
Who can access the data: Anyone
Types of analyses: Any purpose
Mechanisms of data availability: With investigator support
